# Supplementary material for: Targeted next-generation sequencing has incremental value in the diagnostic work-up of patients with suspect pancreatic masses; a multi-center prospective cross sectional study
Source: PLoS One. 2023 Jan 25;18(1):e0280939. doi: 10.1371/journal.pone.0280939 (PMC9876380; doi:10.1371/journal.pone.0280939)
Supplement: S1 File — (PDF) [file pone.0280939.s001.pdf]

Variables Achterberg et. al., 2022

For inquiries contact the corresponding author
J.S.D. Mieog, MD PhD
j.s.d.mieog@lumc.nl
Department of surgery
Leiden University Medical Center
Leiden, the Netherlands

Age	Numeric	2.2|Age at MDO1 [calculated]	None
Sex	Numeric	1.2|Gender	{1, male; 2, female}
Reason_NGS	Numeric	2.25|Reason Next Generation Sequencing	{1, pathologist inconclusive; 2, radiologist inconclusive; 3, cytology inconclusive; 4, MDT unsure; 5, neoadjuvant/LAPC}...
MDO1_date	Date	2.1|Date of MDO1	None
CA19.9	Numeric	2.3|CA19.9 result	None
Radiology	Numeric	2.4|Radiological diagnosis	{1, malignant; 2, benign; 3, cystic lesion; 4, inconclusive}
FNA_brush	Numeric	2.6|Cytology	{1, FNA; 2, brush}
Cytology_result	Numeric	2.8|Morphologic assessment on cytology	{1, dysplasia; 2, atypia; 3, normal; 4, inconclusive}
MDO1_specific_diagnosis	Numeric		{1, pancreatic carcinoma;2, papil carcinoma; 3, distal cholangio carcinoma; 4, IPMN malignant; 5, IPMN benign; 6, autoimmune pancreatatis; 7, pancreatitis; 8, other benign; 9, MCN; 10, pseudocyst}
MDO1_diagnosis	Numeric	2.10|Clinical diagnosis	{1, malignant; 2, benign; 3, cystic lesion; 4, other malignancy; 8, other}
MDO2_diagnosis	Numeric	3.10|Diagnosis after integration of NGS	{1, malignant; 2, benign; 3, cystic lesion; 4, other malignancy; 8, other}
MDO1_correct	Numeric		{0, incorrect; 1, correct}
MDO2_correct	Numeric		{0, incorrect; 1, correct}
MDO2_specific_diagnosis	Numeric		{1, pancreatic carcinoma;2, papil carcinoma; 3, distal cholangio carcinoma; 4, IPMN malignant; 5, IPMN benign; 6, autoimmune pancreatatis; 7, pancreatitis; 8, other benign; 9, MCN; 10, pseudocyst}

Actual_specific_diagnosis	Numeric	4.9|Actual diagnosis: malignant	{1, pancreatic carcinoma;2, papil carcinoma; 3, distal cholangio carcinoma; 4, IPMN malignant; 5, IPMN benign; 6, autoimmune pancreatatis; 7, pancreatitis; 8, other benign; 9, MCN; 10, pseudocyst}
MDO1_treatment	Numeric	2.19|Treatment plan after clinical diagnosis	{1, exploration + resection; 2, neoadjuvant therapy; 3, palliation; 4, follow-up (benign); 5, repeat EUS FNA of brush}
MDO2_treatment	Numeric	3.19|Treatment plan after integration of NGS	{1, exploration + resection; 2, neoadjuvant therapy; 3, palliation; 4, follow-up (benign); 5, repeat EUS FNA of brush}
Actual_diagnosis	Numeric	4.7|Actual diagnosis	{1, malignant; 2, benign; 3, cystic lesion; 4, other malignancy; 8, other}
NGS_contribution	Numeric	3.25|Contribution of NGS	{1, confirmative; 2, change of treatment plan; 3, no value}
NGS_concl	Numeric	3.6|NGS conclusion	{1, high grade dysplasia [HGD]; 2, low grade dysplasia [LGD]; 3, no pathologic variation; 9, inconclusive}
Actual_treatment	Numeric	4.1|Actual performed treatment	{1, exploration + resection; 2, neoadjuvant therapy; 3, palliation; 4, follow-up (benign); 5, repeat EUS FNA of brush}
NGS_correct	Numeric	4.16|NGS correct	{0, no; 1, yes}
Treatmentplan_correct	Numeric		{0, no; 1, yes}
MDO1_neoadj_treatment	Numeric	2.21|Type of neoadjuvant therapy after clinical diagnosis	{1, FOLFIRINOX; 8, other}
MDO1_palliation_reason	Numeric	2.23|Reason palliation treatment after clinical diagnosis	{1, LAPC; 2, metastase; 3, unfit for surgery/wish patient}
MDO2_date	Date	3.1|Date of MDO2	None
Days_MDO1_MDO2	Numeric	3.2|Time between MDO1 and MDO2	None
NGS_representative	Numeric	3.4|Representative material for NGS	{0, no; 1, yes}
NGS_technical_succes	Numeric	3.5|NGS technically successful	{0, no; 1, yes}
nr_path_var	Numeric		None
Gen1	Numeric	3.8|Gene pathologic variant	{1, KRAS; 2, BRAF; 3, GNAS; 4, TP53; 5, APC; 6, ATM; 7, CDKN2A; 8, SMAD4; 9, CTNNB1; 10, PIK3CA}
Gen1_exon	String	3.8|Gene pathologic variant	None
Gen1_freq	Numeric	3.8|Gene pathologic variant	None

Gen1_cov	Numeric	3.8|Gene pathologic variant	None
Gen2	Numeric	3.8|Gene pathologic variant	{1, KRAS; 2, BRAF; 3, GNAS; 4, TP53; 5, APC; 6, ATM; 7, CDKN2A; 8, SMAD4; 9, CTNNB1; 10, PIK3CA}
Gen2_exon	String	3.8|Gene pathologic variant	None
Gen2_freq	Numeric	3.8|Gene pathologic variant	None
Gen2_cov	Numeric	3.8|Gene pathologic variant	None
Gen3	Numeric	3.8|Gene pathologic variant	{1, KRAS; 2, BRAF; 3, GNAS; 4, TP53; 5, APC; 6, ATM; 7, CDKN2A; 8, SMAD4; 9, CTNNB1; 10, PIK3CA}
Gen3_exon	String	3.8|Gene pathologic variant	None
Gen3_freq	Numeric	3.8|Gene pathologic variant	None
Gen3_cov	Numeric	3.8|Gene pathologic variant	None
Gen4	String	3.8|Gene pathologic variant	None
Gen4_exon	String	3.8|Gene pathologic variant	None
Gen5	String	3.9|Specification other gene(s)	None
Gen5_exon	String		None
MDO2_neoadj_treatment	Numeric	3.21|Type of neoadjuvant therapy with NGS	{1, FOLFIRINOX; 8, other}
MDO2_palliation_reason	Numeric	3.23|Reason palliation treatment with NGS	{1, LAPC; 2, metastase; 3, unfit for surgery/wish patient}
NGS_contribution_specify	String	3.27|Additional notes MDO2	None
Reason_palliation	Numeric	4.5|Reason actual palliation treatment	{1, LAPC; 2, metastase; 3, unfit for surgery/wish patient}
Given_neoadj	Numeric	4.3|Type of actual given neoadjuvant therapy	{1, FOLFIRINOX; 8, other}
NGS_not_correct_reason	String	4.17|Reason NGS not correct	None
Second_opinion_cytology	Numeric	4.19|Second opinion comparision	{1, confirmative; 2, change of treatment plan; 3, no value}
Second_opinion_assessment	Numeric	4.20|Second opinion cytology	{1, dysplasia; 2, atypia; 3, normal; 4, inconclusive}
Notes	String	4.23|Additional notes Follow-up	None
